# Antioxidant *N*‐acetylcysteine as an intervention for neuropsychiatric symptoms in individuals with vascular mild cognitive impairment

**DOI:** 10.1002/alz70857_106844

**Published:** 2025-12-24

**Authors:** Alan (Jing Chiuan) Peng, Yejin Kang, Damien Gallagher, Nathan Herrmann, Zahinoor Ismail, Sandra E. Black, Alex Kiss, Paul I. Oh, Joel Ramirez, Walter Swardfager, Kate Survilla, Ethan Mah, Danielle Vieira, Krista L Lanctôt

**Affiliations:** ^1^ University of Toronto, Toronto, ON, Canada; ^2^ Sunnybrook Research Institute, Toronto, ON, Canada; ^3^ Sunnybrook Health Sciences Centre, Toronto, ON, Canada; ^4^ University of Calgary, Calgary, AB, Canada; ^5^ Hotchkiss Brain Institute, Calgary, AB, Canada; ^6^ O’Brien Institute for Public Health, Calgary, AB, Canada; ^7^ Toronto Rehabilitation Institute, University Health Network, Toronto, ON, Canada; ^8^ KITE Research Institute, University Health Network, Toronto, ON, Canada

## Abstract

**Background:**

Neuropsychiatric symptoms (NPS) are non‐cognitive changes that often co‐occur with mild cognitive impairment (MCI) and independently increase the risk of progression to dementia. Of the NPS, apathy is a particularly strong risk factor. Oxidative stress and vascular risk factors of dementia have been implicated together to contribute to neurodegeneration. We previously found that oxidative stress biomarkers in individuals with vascular MCI (vMCI) were associated with apathy, suggesting antioxidants may be potential treatments. Here, we explored the efficacy of antioxidant *N*‐acetylcysteine (NAC) as a treatment for NPS in individuals with vMCI.

**Method:**

This is a secondary analysis from a double‐blind, RCT (“MOVE‐IT”) that was conducted on exercising older adults with vMCI (cardiovascular diseases and ≥1 SD below the norm in executive function, processing speed, memory, or working memory). Participants were recruited from a cardiac rehabilitation program. Over 24 weeks, participants received either 2400mg of NAC or placebo as treatment. Neuropsychiatric assessments were taken at weeks 0, 12, and 24. The Mild Behavioural Impairment Checklist (MBI‐C) was used to capture five main domains of pre‐dementia NPS: apathy, emotional dysregulation (ED), impulse control, social inappropriateness, and psychosis.

**Result:**

All 59 participants (*n* = 29 NAC, *n* = 30 Placebo, 69% male, mean[SD]: age=67.6[7.7], BMI=27.4[5.5], MoCA=22.5[3.2]) provided MBI‐C data for this analysis. Zero‐inflated Poisson mixed models with subject as random effect were used to address the high proportion of participants without NPS and to fit the incremental severity scores in each MBI‐C domain. Covariates included age, sex, and number of cardiovascular risk factors (hypertension, coronary artery disease, hypercholesterolemia, dyslipidemia, type‐2 diabetes, > 5‐year smoking history, obesity). Significant treatment effects over time were observed for apathy (Baseline: 56% apathy+, β[SD] = –1.35[.415], z = –3.26, *p* = .001) and ED (Baseline: 54% ED+, β[SD] = –.731[.306], z = –2.03, *p* = .043) scores at week 12. On average, scores decreased more in the NAC group (mean[SD]: apathy = –1.56[3.27], ED = –2.04[4.20]) than in the placebo group (mean[SD]: apathy = –0.296[1.66], ED = –0.481[1.67]) after 12 weeks. No treatment effects were observed for the other domains.

**Conclusion:**

The findings support NAC as a potential treatment for apathy and emotional dysregulation in individuals with vMCI and at risk of dementia, thereby reducing associated burdens.

